# Adaptive Antiretroviral Therapy Adherence Interventions for Youth Living With HIV Through Text Message and Cell Phone Support With and Without Incentives: Protocol for a Sequential Multiple Assignment Randomized Trial (SMART)

**DOI:** 10.2196/11183

**Published:** 2018-12-20

**Authors:** Marvin E Belzer, Karen Kolmodin MacDonell, Samiran Ghosh, Sylvie Naar, Julie McAvoy-Banerjea, Sitaji Gurung, Demetria Cain, Carolyn A Fan, Jeffrey T Parsons

**Affiliations:** 1 Children’s Hospital Los Angeles Los Angeles, CA United States; 2 Department of Family Medicine and Public Health Sciences Wayne State University School of Medicine Detroit, MI United States; 3 College of Medicine Florida State University Tallahassee, FL United States; 4 Hunter College Center for HIV Educational Studies and Training New York, NY United States

**Keywords:** adaptive clinical trial, antiretroviral therapy, medical adherence, cell phone, HIV, mHealth, text messaging, adolescent

## Abstract

**Background:**

Youth living with HIV (YLH) aged 13 to 24 years made up over a fifth (21%) of new HIV diagnoses in 2016, yet only 27% of YLH are virally suppressed. YLH have been shown to be poorly adherent to antiretroviral therapy (ART); however, there has been limited research investigating how to increase adherence in YLH. Mobile health (mHealth) interventions may be one promising way to do this.

**Objective:**

This study (ATN [Adolescent Trials Network] 144 SMART) aimed to compare adaptive interventions that could increase ART adherence in YLH aged 15 to 24 years. This includes mHealth initiatives, the tapering of interventions, and the use of incentives. Cost-effectiveness of sequencing the interventions without incentives before providing incentives and the savings on societal costs due to suppressed viral loads will be determined. This protocol is part of the ATN Scale It Up program described in this issue by Naar et al.

**Methods:**

This study uses a Sequential Multiple Assignment Randomized Trial design. Approximately 190 participants are being recruited, enrolled, and randomized to either cell phone support or text message support. Both intervention groups receive 3 months of intervention, followed by a second randomization based on response to the intervention. Responders test tapering their intervention, and nonresponders test receiving incentives.

**Results:**

Data collection for this study is projected to begin in August 2018 and last until June 2020.

**Conclusions:**

This is an innovative study, particularly in terms of population, intervention types, focus on cost-effectiveness, and recruitment. This study could be particularly effective in improving adherence in YLH while reducing long-term individual and societal costs.

**Trial Registration:**

ClinicalTrials.gov NCT03535337; https://clinicaltrials.gov/ct2/show/NCT03535337 (Archived by WebCite at http://www.webcitation.org/74alXb92z)

**International Registered Report Identifier (IRRID):**

PRR1-10.2196/11183

## Introduction

### Adherence to Antiretroviral Therapy in Youth Living With HIV

Adherence to antiretroviral therapy (ART) is a critical factor contributing to low rates of viral suppression among youth living with HIV. At the end of 2015, there were approximately 60,300 youth living with HIV (YLH) aged 13 to 24 years in the United States [1], and this number continues to increase. In 2016, YLH made up 21% of new HIV diagnoses—the equivalent of 8451 new cases in that year alone [2]. National treatment guidelines recommend initiating treatment with ART as soon as an individual is ready [3]. Even with simpler, more potent, and better-tolerated medications, viral suppression is difficult to achieve in youth. Only 41% of YLH in 2014 received HIV-related care, 31% were retained in care, and 27% were virally suppressed, the lowest percentage of any age group [1].

Adherence to ART is critical to sustain health, reduce transmission [4], and minimize the development of ART resistance [5]. Nonadherence has both public health implications and personal health risks. High viral load (VL) increases the likelihood of viral transmission [6], and nonadherent individuals may be more likely to transmit drug-resistant strains of the virus [7]. Conversely, treatment adherence decreases the pool of infectious individuals when condom use is poor, even among youth aware of their HIV status [8].

The Centers for Disease Control and Prevention (CDC) released a statement in 2017 that further underscored the importance of ART adherence and VL suppression. The CDC’s statement acknowledged that individuals living with HIV who have an undetectable VL are not able to transmit the HIV virus [9]. This is commonly known by the slogan launched by the Prevention Access Campaign: Undetectable=Untransmittable, or U=U [10], and has ushered in a new era of treatment as prevention for HIV as well as contributed to reduced stigma for those living with HIV [11].

Youth, in particular, are frequently shown to be poorly adherent to ART [12-15]. In a study with ethnic and racial minority YLH, 39% reported suboptimal adherence [15]. In a recent review, only 51% of youth on ART achieved viral suppression of less than 400 copies/mL [16]. Data from the largest cross-sectional study to date found that only 37% of perinatally infected and 27.1% of behaviorally infected youth were virologically suppressed [17]. Of the YLH in the study, 30.5% reported engaging in unprotected sex in the last 90 days and 74.4% had detectable viremia. In addition, young men who have sex with men who have detectable VLs have been found to have higher rates of serodiscordant, condomless anal sex than those with undetectable VLs [18]. Finding effective adherence interventions for YLH to reduce HIV transmission is a public health imperative, and improved ART adherence is a particularly promising avenue.

A growing body of research has explored predictors of adherence to ART in YLH. Depression and anxiety have been consistently associated with poor adherence to ART. Adherence should be considered within the broader contextual issues present in the lives of youth, such as HIV stigma and disclosure, peer relations, and mental health and substance use [13,19,20]. Ethnic or racial minority status [15,21,22], lack of social support [15,22,23], and low self-efficacy [15,24] have also been associated with poor medication adherence in youth living with HIV. Poor medication adherence has been linked to financial and structural challenges such as housing instability [25] and lower level of education [26]. MacDonell et al [15] explored social cognitive predictors of ART adherence in a large, multisite sample of racial and ethnic minority YLH. Results indicated that social support, self-efficacy, psychological symptoms, and substance use were predictors of adherence. Taken together, this body of literature suggests that adherence interventions offering social support and promoting self-efficacy, while aiding with problem-solving of contextual barriers, may be beneficial.

### Interventions for Increasing Antiretroviral Therapy Adherence

Although it has been abundantly clear for over two decades that adherence to ART is a critical problem, the field has identified only a limited number of modestly successful interventions for YLH, including motivational interviewing [27,28], directly observed therapy [29], multisystemic therapy [30], and cell phone reminder calls [31,32]. These failed to demonstrate lasting impact on VL beyond the intervention. All the interventions, except the cell phone reminder pilot studies [31,32], require in-person sessions, which may be difficult for nonadherent youth to complete. Interventions that reach youth more frequently, using modern and youth-friendly means of intervention delivery such as cell phones, may hold promise for improving both short- and long-term adherence.

Cell phones may be a powerful tool for HIV prevention and treatment intervention [33]. Interventions delivered via cell phone may offer an advantage over traditional, in-person interventions in cost, flexibility, and ease of adapting the intervention to the participant. Our pilot study found that daily phone call reminders, with incentives for 80% intervention adherence, were both acceptable and feasible for nonadherent youth [32]. Cell phone intervention delivery allows for tapering of the frequency of calls or texts in response to the needs of each participant, which could help sustain the impact of the intervention over a longer period of time for a lower cost. However, few interventions utilizing cell phones as a stand-alone mode of intervention (vs as part of a larger intervention) have been tested, and even fewer have targeted YLH.

Electronic reminders, particularly text messages, have been found in the short term to impact health behavior, including medication adherence of adults with a range of chronic conditions [34,35]. Text message adherence reminders have demonstrated some success in adults with HIV in Africa [36,37]. Recent studies have demonstrated the efficacy of text messages for adherence support and retention in care among US adolescents and young adults living with HIV, although the majority of studies were pilot studies [38,39]. An uncontrolled pilot study of 25 moderately nonadherent youth aged 14 to 29 years (baseline adherence=75%) using personalized, interactive, daily text messages demonstrated significant improvements in self-reported adherence in a 24-week intervention but lacked the power to detect VL changes [40]. Building on this pilot work, a randomized, controlled crossover study [41] utilizing personalized text messages in moderately nonadherent (missed 1+ dose in the last week or 4+ in the last month) youth aged 16 to 29 years found significant improvement in those reporting >90% adherence at 6 months compared with controls (60.5% vs 51%); improved adherence was maintained at 12 months [41].

Although interventions using text messaging to improve adherence in YLH show promise, cell phone–delivered interventions are not limited to text messages. Cell phone–delivered interventions that use voice calling may offer some of the advantages of text messaging (eg, youth-friendly delivery and modern applicability) but retain more of the important elements of human interaction (eg, social support and alliance). A recent qualitative study supports the use of cell phones as a strategy to maintain adherence to antiretroviral refill appointments at a public HIV clinic in Nigeria [42]. A pilot of cell phone support with incentives (CPS-I) in a cohort of highly nonadherent youth demonstrated significant improvements in adherence and VL during the 24-week intervention and 24-week postintervention [43]. Although promising, this study was small, and 7 of the 19 intervention subjects were unable to adhere to the strict intervention requirements [43].

### Design Base

This study utilizes a Sequential Multiple Assignment Randomized Trial (SMART) design. SMART designs have numerous advantages over traditional trial designs. These are among the newest generation of improved clinical trial designs and methods and are used to inform the development of adaptive treatments or interventions [44,45]. This design is a cost-effective and methodologically rigorous way to maximize clinical utility and real-world implementation in the resulting adaptive intervention. Adaptive interventions are interventions in which the type or dosage of the intervention (eg, number of texts sent) is adjusted based on participant characteristics or response (eg, VL). Thus, an adaptive intervention allows treatment to be tailored to the specific needs of each participant. A SMART design involves multiple intervention stages, with each stage corresponding to one of the critical decisions involved in the adaptive intervention.

### Theoretical Base

The intervention is guided by the conceptual model of supportive accountability [46]. This model was developed to guide research into human support components of mobile health (mHealth) interventions. The model is based on the premise that human support increases adherence through accountability to a coach (in the intervention, an adherence facilitator or AF) who is perceived as trustworthy, knowledgeable, and benevolent. Accountability should involve clear, process-oriented expectations that the patient is involved in determining (eg, reporting adherence, problem-solving). The effect of accountability may be moderated by patient motivation so that patients with higher intrinsic motivation may actually require less support.

The process of support is also mediated by the mode of communication (eg, phone, text messages, and computer), with different advantages and disadvantages for each mode. There is evidence that “lean media,” or those modes of communication with less face-to-face contact and fewer visual social cues, may be associated with more positive, even idealized, attributions of communication partners. This is because people tend to form stronger impressions based on more limited social and interpersonal cues [47]. Interactions via lean media, including CPS and text messages, have the potential to foster social accountability toward improved adherence.

Multiple studies have demonstrated that social support is a strong predictor of good adherence to ART [43,48-50], and retention to HIV care is predicted by clients’ perceptions of providers as engaging and validating [51]. Although overall social support was predictive in these studies, specific aspects such as instrumental support (ie, practical assistance) and informational support (advice or problem-solving) were found to be predictive of adherence in adults with HIV [50]. The content of conversations in the CPS study [43] is designed to validate the importance of adherence, prompt problem-solving (through informational support), and provide instrumental assistance (through referrals) to address barriers as they emerge. This intervention utilizes social support constructs to provide tailored conversations to improve both short- and long-term adherence. Social support theory also suggests that an ongoing alliance could be protective against depression, substance use, and general stress [52-55].

### Cost-Effectiveness

It is increasingly important in the face of competing demands for health care resources to establish not just the efficacy of interventions but also their relative economic value. The National Institutes of Health (NIH) established cost-effectiveness analysis as a key priority in 2015 [56]. By integrating cost-effectiveness analysis into a SMART trial, we can simultaneously determine not only the most effective sequences but also whether the added costs of potentially more effective sequences are worthwhile. Within an implementation science framework, costs include those associated with executing implementation strategies as well as those associated with service delivery as uptake changes [57]. Understanding the potential barriers and facilitators to intervention sequences for widespread implementation can provide additional critical information about treatment sequences. Many have argued that the science-practice gap is inflated by the bias toward step-wise progression of research from development, to efficacy, to effectiveness, to implementation, and that hybrid designs can maximize clinical implementation earlier in this developmental process [58]. Thus, we can greatly enhance the potential for intervention scale-up by comparing interventions in real-world settings and identifying effective sequences of treatments and their cost-effectiveness ratio while simultaneously studying the context of implementation in a Hybrid 1 Effectiveness-Implementation trial [58,59].

In addition, helping people living with HIV maintain an undetectable VL is in itself a way to reduce sexual transmission of HIV, as highlighted by U=U. Therefore, not only is this intervention a potentially cost-effective way to achieve VL suppression in YLH, but it may also prevent future cases of HIV transmission to those who are HIV negative. This potentially saves the health care system hundreds of thousands of dollars per person. A recent study found that the medical cost saved from preventing a single HIV infection is US $229,800 [60]. This rises to US $338,400 if those living with HIV entered into the care cascade early on and stayed in care [60]. This figure grows higher when taking into account secondary infections avoided and societal costs saved (eg, social services and housing, patient and family time, productivity, physical tolls, and emotional distress) [60]. It is clear that VL suppression is a key part of saving medical costs related to HIV care, both for the patient themselves and for the public.

### Aims

The aim of this study is to test an adaptive adherence intervention, which utilizes 2 mHealth intervention designs, in an effort to promote adherence to ART and maintain VL suppression in YLH from across the United States. Both interventions are delivered remotely, utilizing a central research center. In addition, this study aims to increase understanding of the context for wide-scale implementation of this type of intervention as well as to understand the benefit of incentives for nonresponders and tapering of interventions for responders.

The primary hypotheses are as follows:

1. Youth randomized to CPS will have significantly greater VL suppression (primary outcome) and self-reported medication adherence (secondary outcome) at 24 weeks than those in the SMS text message support group.
2. Nonresponders randomized to CPS with incentives will have significantly greater VL suppression (primary outcome) and self-reported adherence (secondary outcome) than those randomized to SMS with incentives.

Secondary aims include assessing the following concepts, all central to the *Scale It Up* program described in this issue [61], within the different intervention sequences within the SMART design: (1) cost-effectiveness; (2) the 5 components of the Self-Management Model over time (see ATN: Scale It Up overview paper in this issue); and (3) the barriers and facilitation throughout Exploration, Preparation, Implementation, and Sustainment (EPIS) Model phases (identified in ATN 153 EPIS protocol paper in this issue).

## Methods

### Overview of Content and Delivery

This study is part of the Scale It Up program as described in the overview paper in this issue [61] and employs a SMART design with repeated measures (NCT03535337). Approximately 190 YLH participants are being recruited, consented, enrolled, and randomized to either CPS or SMS (short message service). CPS is provided on all weekdays except holidays. Weekend and holiday adherence are assessed at the first CPS contact with the participant following the weekend and/or holiday. SMS is provided every day, including weekends and holidays. Both intervention groups receive 3 months of intervention, followed by a second randomization, which is be based upon response to the intervention.

**Figure 1:**
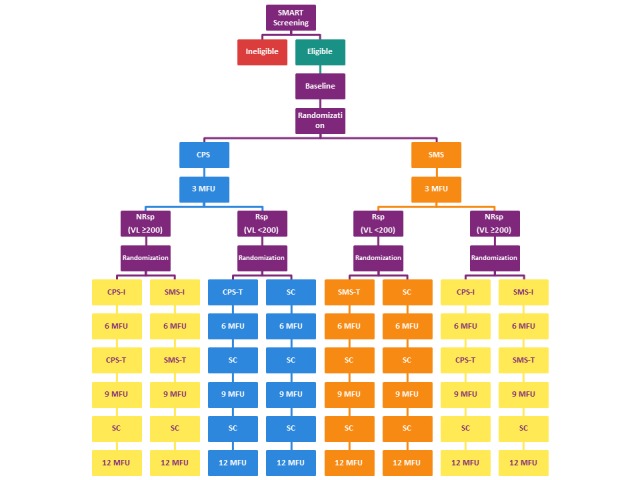
The SMART (Sequential Multiple Assignment Randomized Trial) study design. CPS: cell phone support; CPS-I: incentivized cell phone support; CPS-T: tapered cell phone support; MFU: month follow-up; NRsp: nonresponders; Rsp: responders; SC: standard care; SMS: text messaging support; SMS-I: incentivized text messaging support; SMS-T: tapered cell phone support; VL: viral load.

Participants who respond to the intervention, that is, those with a suppressed VL (<200 copies/mL) in either group, are randomized to receive either 9 months of follow-up without intervention (standard care) or 3 months of tapered intervention (CPS-T or SMS-T twice a week) based on their previous intervention modality, followed by 6 months of follow-up without intervention (standard care; see Figure 1).

Participants who do not respond to the intervention, that is, those with a VL ≥200 copies/mL at the 3-month study visit, are rerandomized to CPS or SMS; however, an incentive is added to both arms. This incentivized CPS or SMS (CPS-I or SMS-I) provides the opportunity to explore the role of incentives with each intervention modality. After 3 months of CPS-I or SMS-I, participants receive 3 months of tapered intervention (CPS-T or SMS-T twice a week) based on their most recent intervention modality followed by 3 months of follow-up without intervention (standard care). Those who are unable to provide a documented VL result at the 3-month time point are considered nonresponsive to the intervention and are rerandomized to an incentive arm.

All participants complete a Web-based, computerized survey, which measures self-reported adherence, substance use, depression, and other mediators and moderators of adherence at baseline and every 3 months thereafter until study completion (12 months for both responders and nonresponders). In addition, a VL result is obtained from participants or their care provider, or they can provide a blood sample for a study-sponsored VL assay.

Participants are compensated US $40 after completing the baseline and each follow-up assessment for a total of US $200 for all study assessments. Participants in the CPS-I or SMS-I arms who reach 75% monthly adherence to calls or text responses receive an additional US $50 for up to 3 months (up to US $150). As all activities are conducted remotely, compensation is provided via electronic gift cards (eg, Amazon, Target, Walmart).

### Recruitment and Eligibility

#### Eligibility Criteria

To be considered eligible for enrollment, an individual must meet the following criteria: (1) a youth living with HIV (aged between 15 years and 0 days and 24 years and 364 days, inclusive, at the time of signed informed consent or assent), (2) willing to provide proof of VL ≥200 copies/mL or blood specimens for HIV VL measurement within 6 months before baseline enrollment, (3) prescribed an ART medication regimen for a minimum of 3 months before eligibility VL, (4) the sole owner of a device capable of sending and receiving calls and text messages, and (5) able to provide consent for the research team to communicate with the participant’s HIV care provider team.

Exclusion criteria include the following: (1) participants whose mental, physical, or emotional capacity does not permit them to complete the protocol as written; (2) inability to understand written or spoken English; or (3) concurrent participant in any behavioral research intervention designed to impact medication or care adherence, as indicated in the screener.

#### Recruitment Methods

**Figure 2:**
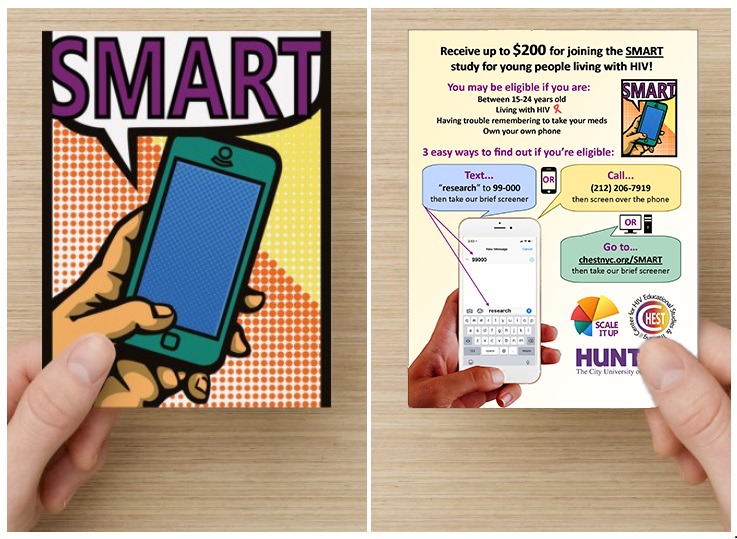
The SMART (Sequential Multiple Assignment Randomized Trial) recruitment card.

**Figure 3:**
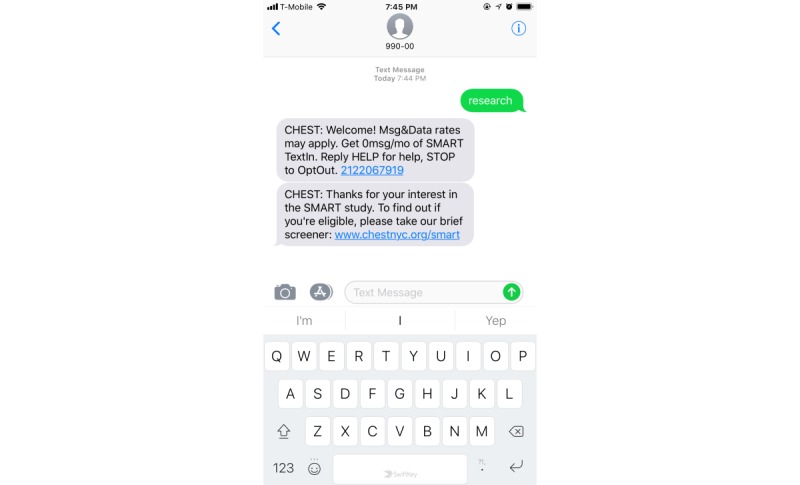
SMART (Sequential Multiple Assignment Randomized Trial) text-in recruitment.

A number of recruitment strategies previously utilized by the Center for HIV/Educational Studies and Training (CHEST) are being implemented [62-64] by the *Scale It Up* Recruitment and Enrollment Center (REC). To ensure the desired sample of 190 participants is reached, both site referrals and national media campaigns are being used. The following are methods through which recruitment takes place: (1) referrals from *Scale It Up* clinical subject recruitment venues (Table 1), (2) social media ad campaigns, (3) geosocial networking data application ads, (4) nationwide flyers and recruitment material distribution (Figures 2 and 3), and (5) indirect recruitment through CHEST Online Master Screener. To date, a nationwide recruitment strategy has not been used for an HIV ART adherence clinical trial.

#### Determining Final Eligibility

**Figure 4:**
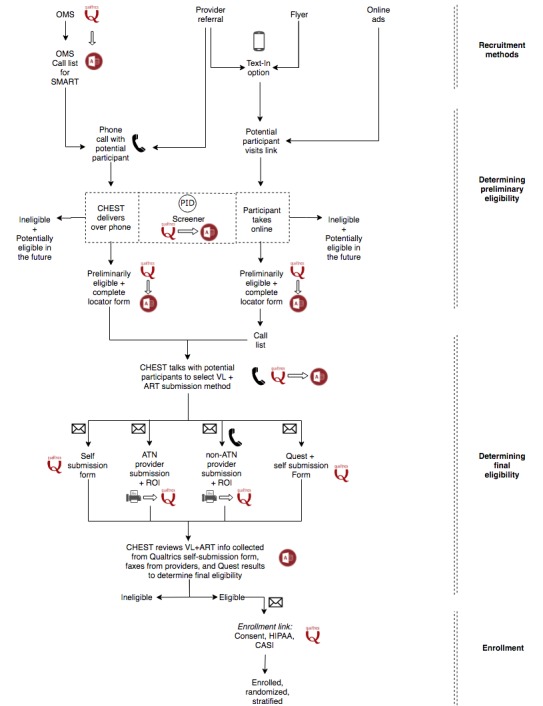
The SMART (Sequential Multiple Assignment Randomized Trial) recruitment flowchart. ART: antiretroviral therapy; ATN: Adolescent Medicine Trials Network for HIV/AIDS Interventions; CASI; computer-assisted self-interview; PID: participant ID; OMS: online master screener; ROI: release of information; VL:viral load.

After potential participants are recruited, participants may complete the study-specific screener online or over the phone (Figure 4). The study screener screens for the inclusion and exclusion criteria for the study. Upon completion of the study screener, interested individuals are informed whether they are preliminarily eligible to participate in the study.

If a participant is preliminarily eligible and interested in participating in the study, the REC staff member discusses several options for submitting proof of VL test results and ART prescription. The staff member explores a number of methods with potential participants, in response to the need to be flexible for the population. The options for submitting proof of an unsuppressed VL (≥200 copies/mL) within 6 months before baseline and of ART prescription within 3 months before the submitted VL test are as follows: (1) self-submission through an online, secure Qualtrics form (eg, uploading a picture of their medication bottle or recent VL results); (2) submission directly by the participant’s health care provider via a release of information; and (3) through study-provided testing at a local Quest site.

**Table 1:** Scale It Up clinical subject recruitment venues.

Site name	Location
Johns Hopkins University	Baltimore, MD
The University of Alabama at Birmingham	Birmingham, AL
SUNY Downstate Medical Center	Brooklyn, NY
Children’s Hospital Los Angeles	Los Angeles, CA
St. Jude Children’s Research Hospital	Memphis, TN
University of Miami	Miami, FL
Children's Hospital of Philadelphia	Philadelphia, PA
University of California San Diego	San Diego, CA
University of South Florida	Tampa, FL
Children’s National Medical Center	Washington, D.C.

It is emphasized to the participants that although the AF can help triage any medical or psychosocial needs that come up during their conversation with the participant, these interactions and access to the AF is not a substitute to continuing to use their main resource for care. For example, if the participant has an acute medical or psychosocial problem or needs an appointment scheduled, they should not call the AF, but instead, use their care site’s working hours or after-hour telephone numbers (as they apply) to get their needs addressed.

A study staff member reviews the potential participant’s VL and ART documentation to determine that they meet the enrollment criteria. A study staff member sends the potential participant the SMART enrollment link, which contains the study consent/assent form, Health Insurance Portability and Accountability Act (HIPAA) Authorization form, and the baseline computerized survey. Upon completion of all portions of the enrollment link, participants are considered enrolled in SMART and are randomized and stratified into their respective intervention arms for the first period of the study.

### Intervention Design

#### Cell Phone Support

Each participant randomized to CPS is assigned a lead and back-up AF. At the time of study entry, the AF and the participant choose a start date and arrange a call time that is after their daily ART dosage time and within office hours. For those participants taking their medication after dinner/before bed, the AF calls in the morning to confirm they took their ART the night before. Although the study allows flexibility in planning for the timing of taking the medication and the phone call that follows is based on the participant’s schedule, the call time is mutually agreeable to the AF and the participant. All calls must take place during the agreed upon time range.

Preferably, calls begin the next Monday following the baseline visit and/or within 2 weeks of study entry. Calls from the AF occur from Monday to Friday, once a day and continue for 3 months, except for major holidays. Although the initial call lasts 10 to 15 min, it is expected that most calls last less than 5 min. AFs take an additional 5 min to document the content of the call. To protect the confidentiality of the participant, the AF confirms that the person who answers the cell phone is the participant enrolled in the study. Voice recognition can be used as the primary confirmation; however, participants are also offered the use of a code word for identification purposes to further protect their privacy. AFs use study cell phones to conduct the daily calls, send/receive texts, and receive voice messages.

If a participant does not answer a prearranged call, the AF leaves a reminder message requesting that the participant calls back within the next 30 min. If a return call is not received within 30 min, the AF repeats the call once at the end of the 30 min. If the participant returns the phone call after more than 30 min have elapsed, the AF conducts the call; however, the delayed call counts as nonadherent to the intervention. If the participant answers but is unavailable to talk, this is counted as nonadherent. If the call is missed due to an issue with the AF, it does not count as nonadherent to the intervention.

On each call, the AF assesses if the participant has taken their medication for that day and if medication was taken during days when calls to the participant were not completed (ie, weekends and holidays). If the participant has not yet taken their ART, the AF waits for the participant to retrieve and take their medication, if available. If the participant usually takes their ART the night before, then the AF does not request that the participant take their medication during the call. If the participant is nonadherent, the AF assesses reasons for nonadherence and engages the participant in brief problem-solving around identified barriers. The AF also discusses any new or ongoing problems in the participant’s life (eg, related to housing, transportation, or food) and provides support in problem-solving to address the issues. In addition, the AF reinforces prioritizing medications, reminds participants about scheduled appointments, and suggests scheduling any relevant referrals (eg, case management, mental health services, and substance use counseling) with their health care providers. Participants needing more intensive assistance are referred to their care team using the information provided on the participant’s locator form, and with the youth’s permission, the AF contacts the care team to share the concerns.

Following the call, the AF completes a checklist about the call. The checklist includes the following: length of the call; time required to reach the participant for intervention purposes; comprehensive record of queries regarding barriers to medication adherence that have arisen during the time between calls; and a place for brief field notes that capture the specifics of medication barriers, advice given, referrals given, and the successful or unsuccessful resolution of barriers when such occurrences arise. The AF also triages any questions or concerns that come up during the course of the call and provides them to the HIV health care provider with the participant’s permission. Any adverse events reported by the participant to the AF are triaged by the REC clinical team and appropriate procedures are followed.

#### Text Message Support

Participants enrolled in the SMS intervention receive daily personalized SMS adherence reminders for 3 months. Participants are able to choose the timing and the wording of the text message to protect confidentiality (eg, have you taken your vitamin today, have you flossed your teeth today). Participants are asked to text back if they did or did not take their ART medications. The texts are sent through Trumpia, a robust, customizable technology to deliver text messages to participants and track their responses. Trumpia is compliant with HIPAA laws as all personal identifiable data are encrypted.

#### Incentives and Tapering

After 3 months of either the CPS or SMS intervention, participants are sorted into new intervention arms depending on their VL. Participants submit proof of VL and complete a computerized survey. Participants whose VL is <200 copies/mL are categorized as “responders,” because they were able to successfully reduce their VL by adhering to their ART medication during the past 3 months. Participants whose VL is ≥200 copies/mL are categorized as “nonresponders.”

All responders are randomized into the tapered intervention arm or into standard care, where no interventions (calls or texts) are made. Those originally in the CPS group receive the CPS tapered (CPS-T) intervention or standard care; likewise, those in the SMS group receive the SMS tapered (SMS-T) intervention or standard care. In the tapered interventions, calls and texts are reduced to 2 days per week. CPS-T and SMS-T last for 3 months until the 6-month follow-up, after which participants transition into standard care. At this point, all responders are in standard care, which continues throughout the 9- and 12-month follow-ups.

Participants who were nonresponders after the first 3 months are randomized to CPS or SMS, but with the addition of an incentive. They receive incentives for text messaging or cell phone support participation. Those participants who answer the cell phone support call or respond to the text messages 75% of the time or more each month receive an additional US $50 during the incentive phase. This is implemented for 3 months until the 6-month follow-up, after which the intervention is tapered to 2 times per week. Those in CPS-I enter CPS-T, and those in SMS-I enter SMS-T. After the 9-month follow-up, participants enter standard care until completion of the study at the 12-month follow-up.

The 3-, 6-, 9-, and 12-month follow-up assessments for all participants include a VL and a computerized survey.

### Training of Interventionists

The AFs are staff members who are not licensed professionals or graduate students who have completed clinical externship training. AFs should (1) be able to interact and engage with youth; (2) be knowledgeable about HIV infection and its treatments, including side effects and their management; (3) be skilled in interpersonal communication and be able to display empathy for the participant; (4) trained in motivational interviewing; (5) maintain a professional relationship and not invade the personal boundaries of the participant; and (6) be trained in providing a link between the participant and their main resource for care, as a means of providing referral services for necessities and counseling, if needed.

All AFs attend a 2-hour webinar designed to familiarize AFs with the purpose of the study, definition of adherence, role of the AF, HIV basics, cultural humility, building rapport and effective communication, legal issues, and protocol review. Following the training, AFs participate in a minimum of 2 telephone role plays during which they practice an initial phone call with a participant and a daily phone call. These training calls last about 30 min, including the role play and feedback. AFs are also encouraged to practice role plays with each other to obtain not only more time in practice but also to experience the role of being the participant during the cell phone support call.

### Fidelity Monitoring

A fidelity evaluation is conducted to assess whether the AF is collecting the correct information and providing an appropriate level of support. This is to ensure fidelity to intervention delivery and to detect areas of the AF training that need to be improved. A clinical coordinator conducts this fidelity evaluation.

All intervention phone calls between the AF and participant in this study are digitally recorded utilizing an amplifier and recorder, and all digital recordings are saved on a secure network for review. The clinical coordinator reviews 20% of all audio files for each AF during the initial 3 months. The audio files are randomly selected from all audio files saved by the AFs. If 90% of reviewed files are found to be adherent to the requirements listed above, the clinical coordinator only reviews 10% of all audio files after the initial 3 months. The recordings are assessed for adherence to the phone call script, advice, referrals, centering of participants in discussion, order of content discussed, and appropriate length of call.

## Results

This study began recruitment in August 2018, and all participant components are projected to end in June 2020.

The primary analysis will be a comparison of the VL suppression rate (primary outcome) between the CPS and the SMS groups at the first stage of randomization (see Study Design). This will be performed using a chi-square test. We will also compare the drop in VL (measured in logarithmic scale with base 10) between the 2 groups. As this is a continuous measure, we will use a 2-sample *t* test to conduct this analysis. We will also compare medication adherence rate (secondary outcome) between the 2 groups using a chi-square test. All the primary analyses will be based on initial assignment to groups, using the intention-to-treat principle. Each of the primary hypotheses will be tested using linear mixed-effects (LMEs) regression analyses [65]. All LMEs will be tested for goodness-of-fit using Wald-type test, which shows satisfactory performance for models with fewer (<5) covariates [66]. For testing primary hypothesis 1, the model will include up to 4 repeated assessments of VL suppression (months 0, 3, 6, and 12) as the dependent variable. For primary hypothesis 2, we will only focus on nonresponders (for both CPS and SMS at stage 1) and compare the VL suppression as dependent variable using a repeated measure LME (months 0, 3, 6, 12). Each LME model will include a random intercept and slope and fixed effects for adherence intervention group, time, and the stratification variables: clinical site, age, and gender. A likelihood ratio test will examine the incremental contribution of the group by time interaction, which represents the interaction of interest for primary hypothesis 1 and primary hypothesis 2, testing for a differential adherence intervention effect over time. The decision rule for each primary hypothesis calls for rejection of null hypothesis if this interaction is statistically significant using the Hochberg step-up alpha adjustment [67]. A site by group interaction will be also examined and included in each model (above) if significant at the .05 level. In addition, likelihood ratio tests will be used to compare the model fit with that having a first-order autoregressive (AR1) covariance structure, as described by Hedeker and Gibbons [65].

We will also conduct similar models as mentioned above, however, predicting adherence to ART (secondary outcome), which is measured at every time point. Each model will be a 2-level model in which time points (level 1) are nested within participants (level 2). This approach accounts for the nonindependence of repeated measurements within individuals. The purpose of the LME-based [68] analysis for the primary aim is to determine which of the first-stage intervention, CPS or SMS, is associated with the most improvement in VL and adherence, regardless of which second-stage treatment participants received. As noted in SMART design, we compare combinations of subgroups (as in 2 specific aims) but not individual subgroups. The study is designed and powered to test the 2 primary hypotheses with 1 primary outcome. As it is customary, secondary and exploratory aims are not powered [69]. However, we expect that data based on SMART design will yield valuable information for hypotheses generation, involving high-quality embedded interventions, which can guide the design of subsequent confirmatory studies.

The first of the secondary aim is to compare the effect of tapering with termination at the second randomization among those who received CPS and those who received SMS and achieved VL <200. We will compare viral suppression rates among the 2 groups (tapering vs termination), followed by a more refined analysis using LME modeling. We will first perform a chi-square test between the 2 viral suppression rates, followed by mixed-effects modeling. The purpose of the second secondary aim is to determine which of the adaptive intervention arms lead to the greatest improvement in VL and adherence over the entire study period. To perform this, we will estimate the viral suppression and adherence rates following each of the 8 embedded interventions and conduct a chi-square test. As both responders and nonresponders are rerandomized, there is no need to use inverse-probability weighting [70]. However, to account for the correlation induced by subjects shared between any 2 embedded interventions, we will use robust (sandwich) SE as in the generalized estimating equations approach [71].

In addition, we will study the moderators of treatment effect. This is a potentially impactful goal, given the gradual but assured paradigm shift in behavioral interventions from “one-size-fits-all” approach to the modern personalized medicine. Potential moderators in the current context are self-reported adherence, substance use, and depression—these can be incorporated in the analysis of the SMART data to deeply personalize the adaptive intervention for future patients. Due to the 2-stage nature of the adaptive interventions, a straightforward regression analysis including potential moderators in the model as interaction terms is not suitable due to the possibility of unmeasured confounding induced by selection bias (also known as “collider-stratification bias”) that can be present in time-varying settings, even in presence of randomization [72]. To avoid this bias, one needs to employ 2 separate regressions corresponding to the 2 stages of SMART and carefully move backward through the stages; such a state-of-the-art approach is known as Qlearning [73]. Each regression will contain interaction terms between the stage-specific treatments and the appropriate stage-specific moderators. If any interactions come out significant, then patient characteristics can be used to deeply tailor the interventions for future patients. This will be performed using the R software package qLearn [74]. As customary, secondary aims are not powered and exploratory in nature.

## Discussion

### Key Innovations

This study is highly innovative in that it focuses on a critical area for intervention—adherence to ART in YLH—and goes beyond what has been done previously in several key ways: (1) it directly compares 2 successful, potentially sustainable, and youth-friendly modes of mHealth youth adherence intervention delivery; (2) the project uses a SMART design to explore a number of key issues surrounding sequencing mHealth interventions in a cost-effective manner that would be practical in clinical settings; (3) the CPS intervention uniquely addresses multiple barriers to ART adherence in real time using a social support framework; and (4) the study explores the clinical and public health costs of sequencing of interventions based on viral suppression and sexual transmission risk.

Technology-based interventions offer tremendous advantages for delivery of brief interventions in terms of reach, ease of replication, anonymity, and cost. They also have an advantage in functionality or how quickly and easily they can be modified for a specific population. mHealth assessments and interventions have been shown to be powerful tools through which brief, targeted interventions can be delivered to people with HIV or at risk of acquiring HIV [75-77]. Texting and cell phone voice calls, in particular, have been linked to improved adherence to ART [41,43,78,79]. However, no study to date has directly compared the use of SMS with CPS for intervention delivery and efficacy. There are currently *no* specific evidenced-based CDC-supported ART adherence interventions for youth. Cell phones are a convenient and culturally relevant mechanism for intervention delivery, and socioeconomically disadvantaged youth and ethnic and racial minority youth report high rates of access and use [43,80-82].

This innovative SMART design [83] not only compares the efficacy of the proposed CPS and SMS interventions but also explores other key issues such as the role of incentives in participant engagement, the impact of tapering intervention frequency, and the potential cost-effectiveness of sequencing the interventions without incentives before providing incentives. This design allows us to explore potential benefits of sequencing our interventions in a way that mimics what would likely happen in a clinical setting (eg, start with the less intensive, less costly interventions, and if that fails, move to the more intensive and costly intervention with incentives). Finally, the design allows us to test an additional secondary aim, whether tapering the interventions and removing incentives will maintain improved adherence in those that reach a suppressed VL (in both arms). All youth adherence intervention studies to date have found that adherence declines once the intervention is over, and tapering an adherence intervention in youth has not yet been done. This will offer clinical sites a less intensive, low-cost option that could be employed indefinitely in highly nonadherent youth.

Another key intervention is the way the study overcomes the numerous barriers to ART adherence (eg, forgetting, not feeling like taking ART, medication reminds me of HIV) [84]. Effective adherence interventions need to address forgetting, but also promote motivation, reinforce positive reasons to take ART as prescribed, and offer solutions to barriers as they arise or change over time [85]. The CPS intervention is based on the model of social accountability, so that cell phones are used to provide instrumental and informational support, foster patient accountability to their AF, and promote real-time problem-focused coping.

A further key innovation is the use of a centralized model for recruitment and for providing the mobile intervention. The centralized model provides a single location in which multiple activities can occur. This can provide cost savings as staff members can be cross-trained to screen, recruit, and provide adherence support. This may be of benefit when case managers in individual clinics may be overloaded with providing direct care to their clients. In addition, nationwide recruitment through social media combined with the distribution of recruitment materials to HIV care providers such as Ryan White-funded clinics may also provide a cost-effective manner to recruit participants when it is getting more difficult to identify and recruit youth with an unsuppressed VL.

Finally, in the clinical setting, cost-effectiveness becomes critical to understanding how to implement sequences and tapering. Each of these adaptive intervention sequences has differing costs, and we will be able to help determine which intervention might be best for which groups (eg, behavioral acquisition vs perinatal acquisition, substance abuse, depression). From a broader public health perspective, it is clear that youth nonadherent to ART are frequently the same patients engaging in other risky behaviors such as condomless anal sex [18]. Thus, a successful intervention such as this one has the potential to lower the risk of HIV transmission. The assessment of sexual risk and viremia will allow us to estimate the cost benefits from reduced HIV transmission for each sequence.

### Limitations

There are some limitations to this study. Participants are not required to verify that they have been on ART for at least 3 months for study enrollment. Although participants must submit proof of an ART medication bottle or prescription, this does not necessarily have to be from at least 3 months ago. It is possible but unlikely that a subject would have inadequate time to suppress VL under 200 copies/mL if use has been less than 3 months.

This study also relies on mHealth intervention methods, specifically through phone calls and text messages, as a way to improve adherence. The requirement to be the sole owner of a device capable of sending and receiving calls and text messages, although necessary for confidentiality, may pose a barrier to some study participants. As a result, this study may not be accessible to all YLH, particularly those who are low income and do not have a mobile device or who may share a mobile device.

Finally, participants are required to be able to communicate in English. This means that YLH who are not yet fluent in English, such as those who have recently immigrated or who live in areas that are primarily non-English speaking, would not be eligible for this study. However, these are populations which could also benefit from ART adherence interventions if they were made to be accessible, and it is plausible that cultural differences in implementation may be missed.

Overall, this dynamic and adaptive study shows great promise. It offers a highly innovative SMART design to assess the efficacy of 2 mHealth interventions to promote adherence to ART.

## Abbreviations

AF: adherence facilitator
ART: antiretroviral therapy
CDC: Centers for Disease Control and Prevention
CPS: cell phone support
CPS-I: cell phone support + incentives
CPS-T: cell phone support tapered
EPIS: Exploration, Preparation, Implementation, Sustainment Model
HIPAA: Health Insurance Portability and Accountability Act
LME: linear mixed-effects
mHealth: mobile health
NIH: National Institutes of Health
REC: Recruitment and Enrollment Center
SMART: Sequential Multiple Assignment Randomized Trial
SMS: short message service (text message support)
SMS-I: text message support + incentives
SMS-T: text message support tapered
VL: viral load
YLH: youth living with HIV
